# Dietary Habits and Lifestyle Factors Associated with Vascular Diseases: A Case–Control Study

**DOI:** 10.3390/healthcare14121739

**Published:** 2026-06-16

**Authors:** Fethi Sada Zekey, Serkan Sahin, Zafer Cengiz Er, Kübra Uyar Zekey, Vugar Ali Turksoy

**Affiliations:** 1 Department of Family Medicine, Faculty of Medicine, Yozgat Bozok University, Yozgat 66100, Türkiyekubra.u.zekey@bozok.edu.tr (K.U.Z.); 2 Department of Medical Pharmacology, Faculty of Medicine, Yozgat Bozok University, Yozgat 66100, Türkiye; serkan.sahin@bozok.edu.tr; 3 Department of Cardiovascular Surgery, Faculty of Medicine, Yozgat Bozok University, Yozgat 66100, Türkiye; erzafer2008@gmail.com; 4 Department of Public Health, Faculty of Medicine, Yozgat Bozok University, Yozgat 66100, Türkiye

**Keywords:** vascular diseases, dietary habits, cooking oils, illness effect, primary care, case–control study, Türkiye

## Abstract

Background/Objectives: Vascular diseases remain a leading cause of global mortality, yet the dietary and lifestyle factors that contribute to them are not fully understood in Central Anatolian populations. This study aimed to quantify the dietary and lifestyle predictors of vascular disease status in a case–control cohort from a tertiary care setting in Yozgat province. Methods: A total of 1452 adults were enrolled from Yozgat Bozok University Research Hospital: Cardiovascular Surgery (CVS; cases, *n* = 720) and Primary Care (PC; controls, *n* = 732). All participants completed a 43-item questionnaire on diet, lifestyle, and sociodemographic characteristics. Binary logistic regression was used to identify independent predictors of case status, with age, sex, education, and income being included in the model. Results: Chronic comorbidities were present in 33.9% of the control group and 80.3% of the case group. Use of olive oil was most strongly associated with control status (odds ratio [OR] = 0.17; 95% confidence interval [CI]: 0.11–0.27), followed by use of butter (OR = 0.25). Paradoxically, fast food (OR = 0.24) and junk food (OR = 0.31) consumption were more frequent among controls. The use of sunflower oil (OR = 2.30), diabetes (OR = 5.22), and elevated serum ferritin (OR = 1.04 per 10 ng/mL) independently predicted a higher likelihood of being in the case group. The model explained 54.8% of the variance (Nagelkerke R^2^ = 0.548). Conclusions: The apparently higher prevalence of unhealthy dietary behaviours among controls is most plausibly attributed to post-diagnosis dietary modification among cases (an ‘illness effect’), underscoring the window for intervention before disease onset. As this case–control design precludes causal inference, these associations are hypothesis-generating. Promoting olive oil and reducing sunflower oil represent practical, culturally feasible dietary targets for cardiovascular risk counselling in Central Anatolia, pending prospective confirmation.

## 1. Introduction

Vascular disorders, which include coronary artery disease, peripheral arterial disease, cerebrovascular disease, and hypertension, affect a significant proportion of the global population and are the main cause of premature death and disability [1]. The associated healthcare burden is enormous: an estimated 18.6 million deaths are attributed to cardiovascular diseases annually, with low- and middle-income countries being disproportionately affected [1,2]. According to the World Health Organization, ischaemic heart disease and stroke together accounted for around 27% of all global deaths in 2019 [3]. In particular, peripheral arterial disease is a chronic condition and a major public health concern. Its risk factors include poor dietary habits, low levels of physical activity, tobacco use, and comorbidities such as hypertension, diabetes mellitus, and hyperlipidaemia [4].

A central tenet of modern cardiovascular disease (CVD) prevention is that key risk factors can be modified. While age, sex, and family history are non-modifiable determinants, behavioural exposures such as dietary quality, physical inactivity, tobacco use, and excess body weight account for over 90% of the attributable risk of myocardial infarction across populations [5]. Notably, while the additional survival benefit of adhering to a healthy lifestyle may diminish with advancing age, prospective evidence suggests that adults who maintain favourable lifestyle habits around the age of 70 are more likely to enjoy better health and functional independence in later life [6]. Nutritional patterns play a particularly prominent role in this context. Dietary and policy frameworks consistently identify the following as the highest-priority evidence-based targets for preventing cardiovascular disease (CVD), diabetes mellitus, and obesity: increased intake of fruits, non-starchy vegetables, nuts, legumes, fish, and minimally processed whole grains; and reduced consumption of red and processed meats, refined carbohydrates, added sugars, and excess sodium [7]. The 2026 American Heart Association scientific statement further affirms that poor diet quality is one of the strongest modifiable determinants of cardiovascular morbidity and mortality, and it provides updated, practical guidance for clinicians [8]. A substantial proportion of preventable cardiovascular disease (CVD) deaths are attributable to tobacco exposure [9], and structured physical activity reduces the risk of all-cause mortality independently of other factors [10].

Physical activity is defined as any bodily movement produced by skeletal muscle contraction that results in energy expenditure above resting levels. Exercise is a planned, repetitive, and purposeful subset of physical activity aimed at improving or maintaining physical fitness. The Mediterranean dietary pattern, centred on olive oil, an abundance of vegetables, moderate amounts of fish and dairy products, and limited amounts of red meat, has the most substantial body of prospective evidence for cardiovascular disease (CVD) prevention across heterogeneous populations [11]. In contrast, diets high in refined carbohydrates, processed meats, and omega-6-rich seed oils have been associated with pro-atherogenic lipid profiles and systemic inflammation [12]. From a clinical and assistive practice standpoint, translating population-level dietary evidence into behavioural change for patients requires attention to nutritional knowledge gaps at the point of care. Studies assessing the nutritional knowledge of patients when they first access diabetes centres reveal significant deficits in understanding the impact of cooking fats, refined carbohydrates, and processed foods on cardiometabolic health. These deficits persist even among patients with established comorbidities [13]. Medical nutrition therapy is an essential but frequently underutilised component of diabetes management. Improving clinician nutrition literacy has been shown to increase the frequency and quality of dietary counselling in primary care settings [14]. In the context of long-term care, food and nutrition care standards are critical yet often neglected levers for the secondary prevention of cardiovascular and metabolic diseases among institutionalised older adults, who bear a disproportionate burden of comorbid hypertension, type 2 diabetes, and heart failure [15].

Despite the extensive literature on this topic, Central Anatolia is a region that has been studied very little. Its food culture is characterised by the widespread use of sunflower oil, a high consumption of red meat and bread, and a comparatively low olive oil intake, which is a pattern that could potentially lead to an increased risk of cardiovascular disease (CVD). Another issue complicating dietary CVD research is the ‘illness effect’: patients who receive a cardiovascular diagnosis often substantially alter their eating habits, introducing reverse causality bias into case–control comparisons. To our knowledge, no previous case–control study has characterised cooking fat preferences, routine biochemical markers, and the illness effect within a single Central Anatolian cohort concurrently. By integrating these elements within one explicitly framed analytical design, the present study addresses a clearly defined regional evidence gap and offers a basis for interpreting dietary associations in a population whose habitual diet diverges substantially from the Mediterranean pattern that dominates the existing prevention literature [7,11].

The primary objective of this study was to use binary logistic regression with adjustment for key sociodemographic confounders to identify independent dietary and lifestyle predictors of vascular disease status in a tertiary care case–control cohort from Yozgat province in Central Anatolia. The secondary objectives were to quantify the contribution of biochemical markers, including serum ferritin, blood urea nitrogen, and free thyroxine, to differentiating cases from controls, and to characterise the ‘illness effect’ as a source of protopathic bias in cross-sectional dietary assessments of patients with established vascular disease.

## 2. Materials and Methods

### 2.1. Study Design and Setting

This hospital-based case–control study was conducted at the Yozgat Bozok University Research Hospital, which is a tertiary referral centre serving a predominantly rural Central Anatolian catchment area. Reporting follows the Strengthening the Reporting of Observational Studies in Epidemiology (STROBE) checklist for case–control studies [16]. Ethical approval was granted by the Yozgat Bozok University Clinical Research Ethics Committee (approval no. 2017-KAEK-189, 28 October 2020), and all procedures were performed in accordance with the Declaration of Helsinki (revised 2013) [17]. The completed STROBE reporting checklist is provided as Supplementary Materials (Table S1).

Inclusion criteria: age of at least 18 years; ability to provide written informed consent; capacity to complete the questionnaire. The ability to provide written informed consent refers to the legal and ethical capacity to give voluntary, documented agreement to participate, as assessed by the recruiting clinician. The capacity to complete the questionnaire refers to the sufficient cognitive and communicative ability to understand and respond to items administered by the research assistant, as confirmed by the assistant. Participants who could not meet these criteria were excluded under the severe cognitive impairment exclusion criterion. Exclusion criteria: active malignancy; pregnancy; severe cognitive impairment; ≥10% missing questionnaire items.

### 2.2. Participants

Adults aged 18 years or over who presented to the Cardiovascular Surgery (CVS) or Primary Care (PC) outpatient department and provided written informed consent were enrolled consecutively. CVS cases had a clinician-confirmed diagnosis of vascular disease (including coronary artery disease, peripheral arterial disease, cerebrovascular disease—such as ischaemic stroke or transient ischaemic attack—and aortic disease), as determined by clinical evaluation, imaging, or operative findings. PC controls presented for routine preventive care or non-cardiovascular complaints and had no current or prior diagnosis of vascular disease.

### 2.3. Sample Size and Power

For sample size estimation, olive oil consumption rates were assumed to be 35% among cases and 60% among controls, based on conservative estimates from the Mediterranean diet literature. A two-sided Pearson’s chi-squared test with α = 0.05 and 80% statistical power indicated that at least 130 participants were required in each group [18,19]. The final sample size (cases *n* = 720, controls *n* = 732) provides over 99% power for this comparison and is sufficient to support a stable logistic regression model with 13 candidate predictors (ratio of approximately 55 cases per variable, well above the conventional minimum of 10:1).

### 2.4. Data Collection

Trained research assistants administered a 43-item, interviewer-administered questionnaire capturing sociodemographic characteristics (e.g., age, sex, occupation, education, income, residence, and marital status), food consumption frequencies, dietary practices, and lifestyle variables (e.g., smoking status, alcohol consumption, and physical activity). The questionnaire is provided as Supplementary Materials (Table S2). Dietary and consumption-related variables (e.g., daily sugar and salt intake, eating speed) were assessed through self-reporting by the participants. For these items, participants rated their own intake subjectively using predefined response options (e.g., ‘none’, ‘low’, ‘normal’, ‘high’) rather than quantitative thresholds. No objective measurements (such as grams per day) or researcher-defined reference values were applied; the categories reflect the respondents’ own perception of their consumption. Physical activity was classified as follows: daily, 3–4 days per week, once per week, or none, based on self-reporting. Information on medication use, including antihypertensives, lipid-lowering agents, and antidiabetics, was not collected via a structured questionnaire. Where available, this information was partially retrieved from clinical records. A pilot study involving 30 volunteers was conducted to assess the internal consistency of the 10-item food consumption subscale (Cronbach’s α = 0.82), indicating satisfactory reliability. Biochemical data were extracted from routine clinical records from the six months prior to enrolment. No additional laboratory investigations were conducted for the purposes of this study. In accordance with standard clinical practice at the study institution, serum samples were obtained following a minimum overnight fasting period of 8–12 h. Analyses were performed on venous blood samples collected between 08:00 and 10:00 in the morning and processed using automated enzymatic and immunoturbidimetric assays on an analyser in the hospital’s central laboratory.

### 2.5. Statistical Analysis

Analyses were performed using IBM SPSS Statistics v23.0 (IBM Corp., Armonk, NY, USA) [20]. Continuous data are presented as the mean ± standard deviation (SD), and categorical data are presented as frequencies and percentages. Distributional normality was assessed using the Kolmogorov–Smirnov test. Independent-samples *t*-tests were used for between-group comparisons of normally distributed data, while Mann–Whitney U tests were used for non-normally distributed data. Pearson’s chi-square test or Fisher’s exact test were used as appropriate, and Kruskal–Wallis was used for non-parametric multi-group comparisons. Listwise deletion was applied to missing values. The complete-case analytical sample comprised all participants with non-missing data on the variables entered into the regression model. Collinearity was assessed using variance inflation factors (VIFs), all of which were below 5.0, indicating acceptable collinearity. The reference categories for all binary and categorical predictors are specified in Section 3.

A binary logistic regression model was fitted, using group membership (CVS = 1, PCU = 0) as the dependent variable. Under this coding, an adjusted odds ratio (OR) above 1 denotes higher odds of case (vascular disease) status, whereas an OR below 1 denotes lower odds of case status and, therefore, an association with the control group; this convention is applied consistently throughout Section 3. Variables that were significant at *p* < 0.05 in the univariate analysis were considered as candidates. Age, sex, educational attainment, and income were forced into the model a priori as potential confounders. The results are presented as adjusted odds ratios (ORs) with 95% confidence intervals (CIs). Model fit was evaluated using the Hosmer–Lemeshow test, and explained variance was quantified using Nagelkerke R^2^. The conventional two-sided significance threshold was *p* < 0.05.

## 3. Results

### 3.1. Sociodemographic Characteristics

A total of 1452 participants were enrolled in the study: 732 controls (PCU) and 720 cases (CVS). Of these participants, 43.3% were male, and 56.7% were female, with an overall mean age of 47.08 ± 14.6 years. Among the 720 CVS cases, the distribution of vascular diagnoses was as follows: coronary artery disease, 542 (75.3%); peripheral arterial disease, 110 (15.3%); cerebrovascular disease, 53 (7.4%); and aortic disease, 15 (2.1%). There were significant differences between the two groups in terms of educational attainment (university graduation was more common in PCU at 32.9%, while primary school graduation was more common in CVS at 60.7%), marital status, urban/rural residence, and employment profile (all *p* < 0.001). The sex distribution was comparable between the groups (*p* = 0.055). The lower total number of participants for the ‘workplace hazard’ variable (N = 872) is due to the exclusion of retirees and housewives, for whom workplace exposure was not applicable at the time of this study. Full sociodemographic data are provided in Table 1.

### 3.2. Chronic Disease Burden and Lifestyle Variables

Non-vascular chronic illness was recorded in 33.9% of the control group and 80.3% of the case group (*p* < 0.001). Diabetes was substantially more prevalent in the CVS group (36.1% versus 5.2%; *p* < 0.001), which is consistent with the well-established link between metabolic syndrome and cardiovascular disease. Asthma was more prevalent among cases (14.8% vs. 1.7%; *p* = 0.010) and their family members (16.4% vs. 3.4%; *p* = 0.019). Controls were more likely to report a family history of hyperlipidaemia (27.6% vs. 6.6%; *p* = 0.002), which may reflect the greater aetiological importance of lifestyle factors compared to genetic predisposition in this cohort. The distributions of chronic diseases are summarised in Table 2.

A prior surgical history and ongoing pharmacotherapy were significantly more prevalent among CVS participants (*p* < 0.001). Current smoking rates were similar between the two groups, but a history of smoking was significantly more prevalent in the PCU group. There was no significant difference in either alcohol consumption or self-reported regular physical activity between groups (both *p* > 0.05).

### 3.3. Food Consumption Frequency

There were significant differences in food consumption frequencies between groups. PCU had a markedly higher red meat intake (≥2×/week) than the control group (57.6% vs. 39.3%; *p* < 0.001). Fast food was essentially absent from the CVS diet. A total of 70.5% of cases reported never consuming it versus 13.6% of controls (*p* < 0.001). Similarly, controls consumed junk food (*p* < 0.001) and canned products (*p* = 0.011) more frequently. By contrast, dairy product intake was significantly higher among cases (68.9% of CVS consumed dairy ≥2×/week vs. 44.1% of PCU; *p* = 0.033). Differences in fruit and vegetables, poultry, eggs, fish, and legumes were apparent but did not reach statistical significance (all *p* > 0.05). Full data appear in Table 3.

### 3.4. Nutritional Habits and Cooking Fat Use

The use of food supplements was over twice as common in the control group (30.5% vs. 11.5%; *p* = 0.010). Controls also reported higher daily sugar consumption (50.8% ‘normal’ versus 24.6% in the CVS group; *p* = 0.002). There were no significant between-group differences in salt use, eating pace, food additive label-reading habits, or mineral water intake (all *p* > 0.05).

Preferences for cooking fats differed substantially. Butter and olive oil were predominantly used by the control group (74.6% and 89.8%, respectively, vs. 26.7% and 33.3% in the CVS group; both *p* < 0.001). In contrast, sunflower oil was the dominant cooking fat among cases (65.0% versus 27.1% in PCU; *p* < 0.001). There was no significant difference between groups for margarine, animal fat, and corn oil. Detailed results are in Table 4.

### 3.5. Biochemical Parameters

Haemoglobin (14.36 ± 1.87 vs. 13.41 ± 1.93 g/dL; *p* = 0.008) and mean corpuscular haemoglobin concentration (MCHC) (33.23 ± 1.46 vs. 32.58 ± 1.20 g/dL; *p* = 0.013) were both higher in the PCU group. In the CVS group, serum ferritin (162.47 ± 213.29 vs. 79.80 ± 75.83 ng/mL; *p* = 0.008), free T4 (1.32 ± 0.24 vs. 1.20 ± 0.18 ng/dL; *p* = 0.006), and BUN (17.96 ± 19.91 vs. 12.27 ± 6.80 mg/dL; *p* = 0.043) were significantly higher. Full data are in Table 5.

### 3.6. Independent Predictors of Vascular Disease Status

Following adjustment for the four forced covariates, binary logistic regression identified 12 independent predictors of case status (CVS). Sex was included in the model but was not statistically significant (*p* = 0.081) (Table 6). The following factors were independently associated with a higher likelihood of being classified as a case: older age (OR = 1.08; 95% CI: 1.04–1.13; *p* < 0.001), low income (OR = 1.75; 1.21–2.54; *p* = 0.004), use of sunflower oil (OR = 2.30; 1.55–3.42; *p* < 0.001), diabetes (OR = 5.22; 3.02–9.02; *p* < 0.001) and an increase of 10 ng/mL in serum ferritin (OR = 1.04; 1.02–1.07; *p* = 0.001).

The independent protective factors associated with a lower likelihood of being classified as a case were as follows: olive oil use (OR = 0.17; 95% CI: 0.11–0.27; *p* < 0.001), butter use (OR = 0.25; 95% CI: 0.16–0.39; *p* < 0.001), consumption of any fast food (OR = 0.24; 95% CI: 0.14–0.40; *p* < 0.001), consumption of any junk food (OR = 0.31; 95% CI: 0.19–0.50; *p* < 0.001), regular consumption of red meat ≥2 times per week (OR = 0.56; 95% CI: 0.37–0.85; *p* = 0.006), use of food supplements (OR = 0.50; 95% CI: 0.30–0.83; *p* = 0.007), and university versus primary school education (OR = 0.48; 0.31–0.74; *p* = 0.001). The model demonstrated satisfactory calibration (Hosmer–Lemeshow *p* = 0.312) and explained approximately 55% of the variance in group membership (Nagelkerke R^2^ = 0.548).

## 4. Discussion

This case–control study compared the dietary patterns, lifestyle factors, and biochemical markers of 720 patients with confirmed vascular disease with those of 732 population-based controls in Central Anatolia. A logistic regression model incorporating 13 variables accounted for around 55% of the variance in group membership. This finding suggests that modifiable behavioural and metabolic factors are the main determinants of vascular disease status in this group, far outweighing the influence of fixed demographic characteristics.

The substantially higher prevalence of diabetes in cases (36.1% versus 5.2%) is consistent with the well-established pathophysiological link between insulin resistance and accelerated atherogenesis, which is mediated by dyslipidaemia, endothelial dysfunction, and low-grade systemic inflammation [12]. Harmonised diagnostic criteria for metabolic syndrome recognise the close mechanistic link between visceral adiposity, hypertriglyceridaemia, and impaired fasting glucose in driving cardiovascular risk [12]. Notably, familial hyperlipidaemia was paradoxically more prevalent in the family histories of controls than cases. This finding is supported by evidence from large genome-wide interaction studies showing that adherence to a healthy lifestyle substantially reduces the cardiovascular risk associated with high polygenic scores [21]. This suggests that, in this Central Anatolian population, environmental factors may be more influential than genetic predisposition in determining clinical vascular disease. Global non-communicable disease burden data also identify behavioural risk factors as the primary drivers of cardiovascular mortality [22,23].

The finding that controls consumed more red meat, fast food, junk food, and tinned products than cases is apparently counterintuitive and demands careful interpretation. These results are biologically counterintuitive and should not be taken as evidence that fast food, junk food, processed foods, or red meat have a protective effect. We attribute this pattern to an ‘illness effect’. Following a clinically significant vascular diagnosis, patients reliably report making substantial dietary changes, reducing their consumption of perceived ‘risky’ foods, and adopting healthier eating practices [24,25]. From an epidemiological standpoint, this represents a protopathic bias that could easily be misinterpreted as evidence of a protective role for these foods. As dietary exposure was measured after the diagnosis of vascular disease, reverse causality is the predominant interpretive constraint on every dietary association reported in this study. Therefore, the directionally counterintuitive odds ratios for processed and ultra-processed foods are best understood as cross-sectional correlates of case–control status rather than as predictors of incident vascular disease. The more constructive clinical message is that behaviour change after diagnosis can be achieved in real-world settings and, more urgently, that primary prevention delivered before the first vascular event would be considerably more effective than modification prompted by disease onset alone [24,25].

The findings regarding cooking fats deserve particular attention. Controls used both olive oil and butter more frequently, whereas sunflower oil predominated among cases. Logistic regression modelling confirmed that the use of olive oil was the strongest dietary factor associated with control status (OR = 0.17; 95% CI: 0.11–0.27), which is consistent with extensive literature on the cardiovascular benefits of the oleic acid, polyphenol, and tocopherol contents of olive oil [10,24]. The association between butter consumption and a lower likelihood of being a case (OR = 0.25) is consistent with meta-analytic data indicating no significant correlation between butter consumption and cardiovascular disease (CVD) incidence or total mortality [25], nor between dairy intake and atherosclerotic cardiovascular events [26,27]. The apparent inverse association of butter should be interpreted with caution, as it likely reflects dietary modifications made after diagnosis rather than a true protective effect. A 2025 umbrella review confirmed that total dairy consumption and yoghurt consumption were both significantly associated with a reduced risk of cardiovascular disease (CVD), and that total dairy consumption and low-fat dairy consumption were both inversely associated with hypertension [28]. The higher reported use of sunflower oil in cases (OR = 2.30) may reflect socioeconomic and cultural patterns rather than an independent causal effect of the oil itself. Sunflower oil is more affordable and plays a more prominent role in Central Anatolian household cooking, particularly in rural and lower-income settings, which were over-represented in the case group. Furthermore, dietary counselling delivered after a vascular diagnosis may have influenced reported cooking fat practices in either direction. Although it has been hypothesised that repeated thermal exposure and reuse of polyunsaturated cooking oils could contribute to vascular risk through the accumulation of lipid oxidation products, this has not been directly demonstrated in our data. As we did not characterise the type of sunflower oil used (refined, unrefined, or high-oleic), its linoleic acid content, or how it was used in cooking, this mechanistic interpretation is presented strictly as a hypothesis for future investigation rather than as a finding supported by the present study. Accordingly, the recommendation to substitute sunflower oil with olive oil should be regarded as hypothesis-generating and requires formal, prospective evaluation before it can be adopted as a population-level policy.

Serum ferritin levels were higher in cases (*p* = 0.008) and were associated with case status in the adjusted model (odds ratio (OR) = 1.04 per 10 ng/mL). However, given the non-standardised sampling conditions, this association should be regarded as exploratory rather than as evidence of an independent biological effect. Where relevant, elevated ferritin levels are most readily interpreted as a marker of chronic, low-grade inflammation in established vascular disease rather than primary iron excess. This is consistent with its well-characterised acute-phase behaviour [12]. The higher BUN in cases (*p* = 0.043) may indicate subclinical renal hypoperfusion; however, it could also reflect differences in hydration, medication, or sampling conditions. Similarly, the higher free T4 concentration in cases (*p* = 0.006) requires cautious interpretation. While there is Mendelian randomisation evidence supporting a causal relationship between subclinical hyperthyroidism and atrial fibrillation risk [29], the present finding cannot establish such a link. Instead, it may reflect enhanced adrenergic tone secondary to established cardiovascular disease or residual measurement heterogeneity [30]. These biochemical findings are therefore hypothesis-generating and require confirmation under standardised sampling conditions.

It is noteworthy that there are no significant differences in current smoking, alcohol use, or physical activity between the groups. However, the significantly higher proportion of ex-smokers among the control group can be interpreted in two ways: it may reflect successful secondary prevention, given that cardiovascular event survivors tend to quit tobacco at higher rates [31], or it may indicate that many of the control group had already benefited from quitting years prior to enrolment in the study. A recent systematic review with GRADE assessment confirmed that smoking cessation substantially improves vascular endothelial function and arterial compliance, with benefits detectable within one month and sustained for at least 24 months [32].

### 4.1. Strengths and Limitations

Strengths. This study enrolled over 1400 participants from two clinically defined groups, enabling statistical adjustment for multiple sociodemographic confounders via logistic regression. Including biochemical data, even if derived from clinical records rather than specific measurements for the study, provides an objective physiological dimension that is rarely available in dietary case–control studies. The explicit framing of the illness effect as an interpretive framework is a methodological strength that distinguishes this report from many comparable studies. The Central Anatolian setting adds geographic novelty and public health relevance to the literature on regional dietary patterns and cardiovascular disease (CVD) risk.

Limitations. There are several limitations that warrant acknowledgement. Firstly, the data collection precludes any causal inference. Self-reported dietary data are susceptible to recall bias and social desirability, and the lack of portion size estimation means that absolute nutrient intake cannot be calculated. While the illness effect is strongly inferred from the data, it remains indirect evidence without a prospective dietary assessment before and after diagnosis. Furthermore, due to the nature of the hospital-based case–control design, it was not possible to establish the temporal sequence of dietary changes relative to disease onset. Consequently, while the attribution of healthier dietary patterns in the case group to a post-diagnosis ‘illness effect’ is clinically plausible, it remains inferential and cannot be definitively separated from potential pre-existing behavioural differences or reverse causality. In addition to reverse causation, the differential recruitment of cases from the Cardiovascular Surgery Unit and controls from the Primary Care Outpatient Clinic introduces selection bias as an alternative potential explanation for the observed dietary differences. The two source populations were not directly comparable with regard to education, income, occupation, residence (urban versus rural), marital status, chronic disease burden, or pharmacotherapy. While the multivariable model adjusted for age, sex, education, and income, residual confounding by socioeconomic, cultural, and lifestyle factors cannot be ruled out.

Structured data on body mass index, smoking intensity, and total energy intake were not collected. Information on the use of medication, including lipid-lowering, antihypertensive, and antidiabetic agents, was not systematically recorded. The data available from routine clinical records were incomplete and could not be reliably quantified. Consequently, residual confounding by these unmeasured factors cannot be ruled out. Physical activity was assessed using broad, frequency-based self-report categories. Data on exercise duration, intensity, and occupational physical activity were unavailable. Therefore, residual confounding related to physical activity patterns cannot be excluded. The dietary instrument did not capture portion sizes, total energy intake, or quantitative nutrient estimates. It was also not a formally validated food frequency questionnaire. A satisfactory Cronbach’s α value only indicates internal consistency and does not establish dietary criterion validity. The duration of dietary habits prior to diagnosis could not be assessed. Information on the composition (refined vs. unrefined, high-oleic), brand, and culinary handling of sunflower oil, including reheating and reuse, was not collected. Therefore, interpretations involving lipid oxidation products should be treated with caution. Furthermore, dietary intake categories (e.g., sugar and salt consumption) were based on participants’ subjective self-assessment rather than standardised quantitative criteria or validated dietary recall methods. Consequently, terms such as ‘low’ or ‘normal’ may have been interpreted differently by different participants, which could introduce reporting bias and limit comparability. Biochemical parameters were only available for a subgroup, and these were drawn from routine records with varying assay dates. Residual confounding factors such as BMI, physical activity intensity, and total energy intake cannot be excluded. Given the number of variables examined, some statistically significant associations may be due to chance; the results should therefore be interpreted with caution and replicated in independent cohorts. Single-centre enrolment limits external validity. Finally, although vascular disease was considered a single outcome, the case group comprised coronary artery disease, peripheral arterial disease, cerebrovascular disease, and aortic disease, each of which has a different risk profile. These conclusions would be substantially strengthened by future prospective, multi-site studies incorporating validated 24 h dietary recalls or objective food biomarkers.

### 4.2. Perspectives for Clinical and Preventive Practice

Despite the methodological limitations of a hospital-based case–control study design, this study’s findings have practical implications for clinical and preventive practices that warrant brief consideration. Firstly, the significant difference in dietary habits between the case and control groups demonstrates how quickly individuals change their eating habits once a vascular diagnosis has been made. This responsiveness presents a clear opportunity for primary care, family medicine, and outpatient cardiovascular clinics to provide structured dietary counselling well before disease onset, particularly in rural and lower-income Central Anatolian communities where sunflower oil predominates and olive oil is comparatively underutilised. Secondly, the observed associations highlight the importance of food knowledge and dietary literacy during the initial clinical consultation. This is consistent with evidence from diabetes care settings, where baseline nutritional knowledge influences long-term metabolic outcomes [33]. Taken together, these findings reinforce the idea that dietary modification is one of the most effective ways of reducing the regional burden of vascular disease, while emphasising that the strength of any such recommendation must be calibrated to the underlying evidence. Thirdly, from a policy standpoint, regionally targeted public health campaigns focusing on affordable Mediterranean-style dietary patterns—particularly the substitution of sunflower oil for olive oil—should be evaluated prospectively as candidate interventions rather than being adopted as established population guidance, based on cross-sectional evidence alone [34]. Fourthly, healthcare professionals, dietitians, and community nurses should be equipped to deliver culturally adapted nutritional counselling that recognises local food culture, household economics, and cooking practices, in line with the broader principles of nutritional care in chronic disease management [35,36]. Taken together, these perspectives reinforce the idea that dietary modification is one of the most effective ways of reducing the regional burden of vascular disease, while emphasising that the strength of any such recommendation must be calibrated to the underlying evidence.

## 5. Conclusions

In this hospital-based case–control study of 1452 adults from Central Anatolia, a multivariable logistic regression model that explained approximately 55% of the variance in group membership (Nagelkerke R^2^ = 0.548) identified modifiable dietary factors, most notably olive oil use (adjusted OR = 0.17; 95% CI: 0.11–0.27) and sunflower oil use (OR = 2.30; 95% CI: 1.55–3.42) together with diabetes (OR = 5.22; 95% CI: 3.02–9.02) and elevated serum ferritin (OR = 1.04 per 10 ng/mL; 95% CI: 1.02–1.07), as the strongest correlates of vascular disease case status after adjustment for age, sex, education, and income. Because dietary exposures were ascertained after diagnosis, these associations are best interpreted as cross-sectional correlates of case status rather than as predictors of incident vascular disease. Consistent with this interpretation, the directionally counterintuitive lower odds of case status associated with fast food (OR = 0.24) and junk food (OR = 0.31) consumption are most plausibly attributable to post-diagnosis behavioural modification (the “illness effect”) rather than to a true protective role of these foods; the present data, therefore, do not support a causal reading of these specific associations. Taken together, the results are best regarded as hypothesis-generating, yet they consistently identify dietary behaviour and the contrast between olive oil and sunflower oil, in particular, as the dominant and potentially modifiable correlate of vascular disease status in this population. Accordingly, the findings support delivering structured dietary counselling and food-literacy interventions before the clinical onset of vascular disease, and they prioritise the substitution of sunflower oil with olive oil as a candidate intervention; the strength of any population-level recommendation must, however, remain calibrated to the cross-sectional nature of the evidence. Future prospective, multi-centre studies incorporating validated dietary assessment instruments, standardised biochemical sampling, and longitudinal follow-ups are needed to establish temporal relationships and to inform region-specific cardiovascular prevention policy.

## Figures and Tables

**Table 1:** Sociodemographic characteristics of participants by group (*n* = 1452).

	Category	PCU (G0) N	PCU %	CVS (G1) N	CVS %	χ^2^	*p*
Gender (*n* = 1452)	Male	298	40.7%	330	45.8%	3.68	0.055
	Female	434	59.3%	390	54.2%		
Occupation (*n* = 1358)	Officer/Civil servant	212	29.0%	71	11.3%	385.63	<0.001 *
	Employee	150	20.5%	24	3.8%		
	Self-employed	74	10.1%	118	18.8%		
	Housewife	161	22.0%	307	49.0%		
	Unemployed	37	5.1%	-	-		
	Retired	12	1.6%	94	15.0%		
	Student	12	1.6%	12	1.9%		
	Farmer	62	8.5%	-	-		
Workplace hazard (*n* = 872)	Less dangerous	50	10.6%	342	85.3%	540.79	<0.001 *
	Dangerous	37	7.9%	12	3.0%		
	Very dangerous	37	7.9%	35	8.7%		
	Do not know	347	73.7%	12	3.0%		
Employment duration (*n* = 899)	0–2 years	99	18.6%	12	3.3%	440.80	<0.001 *
	2–5 years	273	51.2%	23	6.3%		
	5–10 years	74	13.9%	12	3.3%		
	≥10 years	87	16.3%	319	87.2%		
Monthly income (*n* = 1366)	Very low	99	14.8%	94	13.5%	49.12	<0.001 *
	Low	397	59.3%	519	74.6%		
	Middle	112	16.7%	59	8.5%		
	High	62	9.3%	24	3.4%		
Educational attainment (*n* = 1440)	Not literate	-	-	35	4.9%	630.60	<0.001 *
	Literate	12	1.7%	24	3.3%		
	Primary school	62	8.6%	437	60.7%		
	Secondary school	62	8.6%	83	11.5%		
	High school	174	24.2%	83	11.5%		
	College/Vocational	74	10.3%	24	3.3%		
	University graduate	237	32.9%	35	4.9%		
	Postgraduate	99	13.8%	-	-		
Residence (*n* = 1402)	Rural	-	-	177	24.6%	189.66	<0.001 *
	Urban	682	100.0%	543	75.4%		
Marital status (*n* = 1440)	Married	533	74.0%	637	88.5%	84.75	<0.001 *
	Single	150	20.8%	35	4.9%		
	Divorced	12	1.7%	24	3.3%		
	Widowed	25	3.5%	24	3.3%		

* *p* < 0.05 indicates a statistically significant between-group difference. PCU = Primary Care Unit (reference/control group, *n* = 732); CVS = Cardiovascular Surgery Unit (case group, *n* = 720). Between-group comparisons were performed using Pearson’s chi-square (χ^2^) tests for all categorical variables. Cells displaying ‘-’ indicate zero observed counts in that category. Percentages reflect valid responses within each variable; denominators vary due to missing data, as indicated by the sample size in parentheses for each category. Percentages may not sum exactly to 100 due to rounding.

**Table 2:** Distribution of chronic conditions in participants and their family members by group.

Illness	Group	Self N	Self %	*p* (Self)	Family N	Family %	*p* (Family)
Any chronic disease	PCU (G0)	248	33.9%	<0.001 *	633	86.4%	0.373
	CVS (G1)	578	80.3%		578	80.3%	
Diabetes mellitus	PCU (G0)	37	5.2%	<0.001 *	360	50.0%	0.424
	CVS (G1)	260	36.1%		307	42.6%	
Kidney disease	PCU (G0)	12	1.7%	0.338	-	-	0.089
	CVS (G1)	35	4.9%		35	4.9%	
Asthma	PCU (G0)	12	1.7%	0.010 *	25	3.4%	0.019 *
	CVS (G1)	106	14.8%		118	16.4%	
Hyperlipidaemia	PCU (G0)	12	1.7%	0.109	199	27.6%	0.002 *
	CVS (G1)	59	8.2%		47	6.6%	
Psychiatric disease	PCU (G0)	12	1.7%	0.307	-	-	-
	CVS (G1)	-	-		-	-	

* *p* < 0.05 indicates a statistically significant between-group difference. PCU = Primary Care Unit (reference/control group, *n* = 732); CVS = Cardiovascular Surgery Unit (case group, *n* = 720). Between-group comparisons for self-reported and family history of illness were performed using Pearson’s chi-square (χ^2^) tests. ‘Self’ refers to participant-reported personal diagnosis; ‘Family’ refers to first-degree family history as reported by the participant. Cells displaying ‘-’ indicate zero observed counts or no reportable data in that category; corresponding *p*-values are not calculable and are similarly denoted ‘-’. Percentages reflect valid responses within each variable. Percentages may not sum exactly to 100 due to rounding.

**Table 3:** Food consumption frequency across both groups (*n* = 1452).

Food Item	Group	2–3×/Week	1×/Week	1×/Month	Rarely	Never	χ^2^	*p*
Red meat	PCU (G0)	57.6%	42.4%	-	-	-	>16.27	<0.001 *
	CVS (G1)	39.3%	37.7%	9.8%	13.1%	-		
Fish	PCU (G0)	-	16.9%	47.5%	30.5%	3.4%	6.21	0.184
	CVS (G1)	13.1%	24.6%	27.9%	18.0%	16.4%		
Fruit and vegetables	PCU (G0)	57.6%	40.7%	-	-	-	5.26	0.262
	CVS (G1)	75.4%	16.4%	3.3%	1.6%	1.6%		
White meat/poultry	PCU (G0)	37.3%	50.8%	3.4%	5.1%	-	6.25	0.100
	CVS (G1)	65.6%	24.6%	4.9%	4.9%	-		
Milk and dairy	PCU (G0)	44.1%	35.6%	13.6%	3.4%	1.7%	10.49	0.033 *
	CVS (G1)	68.9%	23.0%	3.3%	3.3%	1.6%		
Eggs	PCU (G0)	55.9%	35.6%	6.8%	-	-	5.40	0.145
	CVS (G1)	75.4%	18.0%	4.9%	1.6%	-		
Legumes	PCU (G0)	33.9%	54.2%	8.5%	3.4%	-	6.54	0.088
	CVS (G1)	59.0%	27.9%	6.6%	4.9%	-		
Fast food	PCU (G0)	10.2%	23.7%	27.1%	23.7%	13.6%	>18.47	<0.001 *
	CVS (G1)	1.6%	4.9%	1.6%	21.3%	70.5%		
Junk food	PCU (G0)	23.7%	33.9%	22.0%	13.6%	5.1%	>18.47	<0.001 *
	CVS (G1)	6.6%	4.9%	6.6%	21.3%	60.7%		
Canned food	PCU (G0)	3.4%	10.2%	27.1%	42.4%	15.3%	13.06	0.011 *
	CVS (G1)	4.9%	9.8%	11.5%	18.0%	55.7%		

* *p* < 0.05 indicates a statistically significant between-group difference. PCU = Primary Care Unit (reference/control group, *n* = 732); CVS = Cardiovascular Surgery Unit (case group, *n* = 720). Food consumption frequencies were assessed using a structured five-point ordinal scale (2–3×/week, 1×/week, 1×/month, rarely, never). Between-group comparisons were performed using Pearson’s chi-square (χ^2^) tests across response categories. Cells displaying ‘-’ indicate zero observed counts in that frequency category. Data presented are from a representative subsample; denominators vary by food item due to missing responses. Percentages reflect valid responses within each variable and may not sum exactly to 100 due to rounding.

**Table 4:** Nutritional habits and dietary behaviours by group.

	Response	PCU (G0) N	PCU %	CVS (G1) N	CVS %	*p*
Food supplement use (*n* = 1452)	Yes	223	30.5%	83	11.5%	0.010 *
	No	509	69.5%	637	88.5%	
Daily sugar use (*n* = 1452)	None	-	-	59	8.2%	0.002 *
	Low	199	27.2%	366	50.8%	
	Normal	372	50.8%	177	24.6%	
	High	161	22.0%	118	16.4%	
Daily salt use (*n* = 1452)	None	37	5.1%	71	9.9%	0.087
	Low	211	28.8%	330	45.8%	
	Normal	434	59.3%	248	34.4%	
	Heavily salted	50	6.8%	71	9.9%	
Eating speed (*n* = 1428)	Slow	112	15.3%	224	31.1%	0.657
	Normal	385	52.5%	201	27.9%	
	Fast	223	30.5%	236	32.8%	
	Very fast	12	1.7%	35	4.9%	
Additive label checking (*n* = 1428)	Never	199	27.6%	449	63.3%	0.110
	Sometimes	422	58.6%	71	10.0%	
	Always	99	13.8%	189	26.7%	
Mineral water use (*n* = 1452)	Daily (1–3 btl)	25	3.4%	47	6.5%	0.650
	Daily (4–9 btl)	25	3.4%	12	1.7%	
	Every other day	211	28.8%	47	6.5%	
	1–2×/week	298	40.7%	130	18.0%	
	1–2×/month	174	23.8%	142	19.7%	
	Never	-	-	342	47.5%	
Animal fat use (*n* = 1440)	No	645	88.1%	649	91.7%	0.526
	Yes	87	11.9%	59	8.3%	
Margarine use (*n* = 1440)	No	633	86.4%	649	91.7%	0.365
	Yes	99	13.6%	59	8.3%	
Butter use (*n* = 1440)	No	186	25.4%	519	73.3%	<0.001 *
	Yes	546	74.6%	189	26.7%	
Sunflower oil use (*n* = 1440)	No	533	72.9%	248	35.0%	<0.001 *
	Yes	199	27.1%	460	65.0%	
Corn oil use (*n* = 1440)	No	608	83.1%	637	90.0%	0.270
	Yes	124	16.9%	71	10.0%	
Olive oil use (*n* = 1440)	No	74	10.2%	472	66.7%	<0.001 *
	Yes	658	89.8%	236	33.3%	

* *p* < 0.05 indicates a statistically significant between-group difference. PCU = Primary Care Unit (reference/control group, *n* = 732); CVS = Cardiovascular Surgery Unit (case group, *n* = 720). btl = bottles. Between-group comparisons were performed using Pearson’s chi-square (χ^2^) tests for all variables. Binary variables (food supplement use, animal fat use, margarine use, butter use, sunflower oil use, corn oil use, olive oil use) were compared as dichotomous outcomes. Ordinal variables (daily sugar use, daily salt use, eating speed, mineral water use) were compared across response categories using Pearson’s chi-square; the ordinal trend was not formally tested. Additive label checking was treated as a nominal categorical variable. Cells displaying ‘-’ indicate zero observed counts in that category. Percentages reflect valid responses within each variable; denominators vary as indicated by the sample size in parentheses. Percentages may not sum exactly to 100 due to rounding.

**Table 5:** Haematological and biochemical parameters by group (mean ± SD).

	PCU (G0) Mean ± SD	CVS (G1) Mean ± SD	*p*
WBC (×10^3^/µL)	7.23 ± 1.75	7.41 ± 2.12	0.412
RBC (×10^6^/µL)	5.01 ± 0.51	4.79 ± 0.78	0.063
Haemoglobin (g/dL)	14.36 ± 1.87	13.41 ± 1.93	0.008 *
MCV (fL)	86.12 ± 4.30	85.15 ± 9.10	0.298
MCH (pg)	28.64 ± 2.10	29.23 ± 8.63	0.541
MCHC (g/dL)	33.23 ± 1.46	32.58 ± 1.20	0.013 *
Haematocrit (%)	43.11 ± 4.48	42.02 ± 9.34	0.217
Platelets (×10^3^/µL)	265.37 ± 63.01	281.75 ± 92.99	0.188
TSH (µIU/mL)	2.50 ± 1.50	2.37 ± 1.43	0.091
Free T4 (ng/dL)	1.20 ± 0.18	1.32 ± 0.24	0.006 *
Ferritin (ng/mL)	79.80 ± 75.83	162.47 ± 213.29	0.008 *
Vitamin B12 (pg/mL)	382.82 ± 163.78	374.83 ± 155.92	0.734
Folic acid (ng/mL)	7.46 ± 2.93	8.59 ± 3.95	0.091
AST (U/L)	17.73 ± 5.47	19.10 ± 7.88	0.218
ALT (U/L)	21.10 ± 13.53	22.52 ± 16.60	0.561
Creatinine (mg/dL)	0.82 ± 0.44	1.27 ± 2.06	0.073
BUN (mg/dL)	12.27 ± 6.80	17.96 ± 19.91	0.043 *
Albumin (g/L)	45.65 ± 3.21	44.59 ± 3.96	0.112
Triglycerides (mg/dL)	145.29 ± 87.31	155.93 ± 68.49	0.334
HDL cholesterol (mg/dL)	45.56 ± 11.63	44.81 ± 10.26	0.603
LDL cholesterol (mg/dL)	118.20 ± 37.70	106.28 ± 38.70	0.087

* *p* < 0.05. PCU = Primary Care Unit; CVS = Cardiovascular Surgery Unit. Abbreviations: WBC = white blood cell count; RBC = red blood cell count; MCV = mean corpuscular volume; MCH = mean corpuscular haemoglobin; MCHC = mean corpuscular haemoglobin concentration; TSH = thyroid-stimulating hormone; T4 = free thyroxine; BUN = blood urea nitrogen; AST = aspartate aminotransferase; ALT = alanine aminotransferase; HDL = high-density lipoprotein; LDL = low-density lipoprotein. Independent-samples *t*-test used for normally distributed variables; Mann–Whitney U test for non-normal distributions; significance threshold *p* < 0.05 throughout.

**Table 6:** Independent predictors of vascular disease case status by group.

	B	SE	OR (95% CI)	*p*
Age (years)	0.08	0.02	1.08 (1.04–1.13)	<0.001 *
Sex (female vs. male)	0.31	0.18	1.37 (0.96–1.95)	0.081
Education (university vs. primary)	−0.74	0.22	0.48 (0.31–0.74)	0.001 *
Low income (yes vs. no)	0.56	0.19	1.75 (1.21–2.54)	0.004 *
Red meat ≥2×/week (yes vs. no)	−0.58	0.21	0.56 (0.37–0.85)	0.006 *
Fast food use (any vs. never)	−1.42	0.26	0.24 (0.14–0.40)	<0.001 *
Junk food use (any vs. never)	−1.18	0.25	0.31 (0.19–0.50)	<0.001 *
Olive oil use (yes vs. no)	−1.76	0.24	0.17 (0.11–0.27)	<0.001 *
Butter use (yes vs. no)	−1.37	0.22	0.25 (0.16–0.39)	<0.001 *
Sunflower oil use (yes vs. no)	0.83	0.20	2.30 (1.55–3.42)	<0.001 *
Food supplement use (yes vs. no)	−0.70	0.26	0.50 (0.30–0.83)	0.007 *
Diabetes (yes vs. no)	1.65	0.28	5.22 (3.02–9.02)	<0.001 *
Ferritin (per 10 ng/mL increase)	0.04	0.01	1.04 (1.02–1.07)	0.001 *
Constant	−2.31	0.48	0.10	<0.001 *

Abbreviations: OR, odds ratio; CI, confidence interval; SE, standard error. Reference categories: Sex: male; Education: primary school; Low income: no; Red meat: <2×/week; Fast food use: never; Junk food use: never; Olive oil use: no; Butter use: no; Sunflower oil use: no; Food supplement use: no; Diabetes: no. Age and ferritin are continuous variables (OR per 1-year and per 10 ng/mL increase, respectively). Outcome coded as case (CVS) = 1, control (PCU) = 0; an OR above 1 indicates higher odds of case (vascular disease) status, whereas an OR below 1 indicates lower odds of case status (i.e., an association with control status). * *p* < 0.05.

## Data Availability

The dataset analysed in this study is available from the corresponding author (V.A.T.) upon reasonable request. Data are not publicly archived owing to participant confidentiality requirements.

## Supplementary Materials

The following supporting information can be downloaded at: https://www.mdpi.com/article/10.3390/healthcare14121739/s1, Table S1: STROBE checklist; Table S2: Content and structure of the study questionnaire.

## Abbreviations

The following abbreviations are used in this manuscript: ALT, alanine aminotransferase; AST, aspartate aminotransferase; BMI, body mass index; BUN, blood urea nitrogen; CI, confidence interval; CVD, cardiovascular disease; CVS, Cardiovascular Surgery outpatient unit (case group); Hgb, haemoglobin; HCT, haematocrit; HDL, high-density lipoprotein cholesterol; LDL, low-density lipoprotein cholesterol; MCH, mean corpuscular haemoglobin; MCHC, mean corpuscular haemoglobin concentration; MCV, mean corpuscular volume; OR, odds ratio; PCU, Primary Care Unit/Family Medicine outpatient (control group); PLT, platelets; RBC, red blood cell count; SD, standard deviation; STROBE, Strengthening the Reporting of Observational Studies in Epidemiology; T4, free thyroxine; TSH, thyroid-stimulating hormone; WBC, white blood cell count; WHO, World Health Organization.

## References

[1] (2025). Global Burden of Cardiovascular Diseases and Risks 2023 Collaborators. Global, Regional, and National Burden of Cardiovascular Diseases and Risk Factors in 204 Countries and Territories, 1990–2023. J. Am. Coll. Cardiol..

[2] Roth G.A., Johnson C., Abajobir A., Abd-Allah F., Abera S.F., Abyu G., Ahmed M., Aksut B., Alam T., Alam K. (2017). Global, regional, and national burden of cardiovascular diseases for 10 causes, 1990 to 2015. J. Am. Coll. Cardiol..

[3] World Health Organization (2023). World Health Statistics 2023: Monitoring Health for the SDGs.

[4] Lewandowska A., Filip R., Garczyński W., Lewandowski T. (2026). The influence of dietary habits and physical activity on quality of life of peripheral arterial disease in patients hospitalized at the Department of Vascular Surgery and Transplantation, Medical University in Białystok. Nutrients.

[5] Yusuf S., Hawken S., Ounpuu S., Dans T., Avezum A., Lanas F., McQueen M., Budaj A., Pais P., Varigos J. (2004). Effect of potentially modifiable risk factors associated with myocardial infarction in 52 countries (the INTERHEART study): Case-control study. Lancet.

[6] Zhang Y., Liu X., Wang H., Li S., Yu C., Pei P., Yang L., Chen Y., Du H., Schmidt D. (2026). Associations of lifestyle factors with survival and health statuses in Chinese older adults: A prospective cohort study. Am. J. Prev. Med..

[7] Mozaffarian D. (2016). Dietary and policy priorities for cardiovascular disease, diabetes, and obesity: A comprehensive review. Circulation.

[8] Mozaffarian D., Afshin A., Benowitz N.L., Bittner V., Daniels S.R., Franch H.A., Jacobs D.R., Kraus W.E., Kris-Etherton P.M., Krummel D.A. (2012). Population approaches to improve diet, physical activity, and smoking habits: A scientific statement from the American Heart Association. Circulation.

[9] Arnett D.K., Blumenthal R.S., Albert M.A., Buroker A.B., Goldberger Z.D., Hahn E.J., Himmelfarb C.D., Khera A., Lloyd-Jones D., McEvoy J.W. (2019). 2019 ACC/AHA guideline on the primary prevention of cardiovascular disease. Circulation.

[10] Paluch A.E., Boyer W.R., Franklin B.A., Laddu D., Lobelo F., Lee D.-C., McDermott M.M., Swift D.L., Webel A.R., Lane A. (2024). Resistance exercise training in individuals with and without cardiovascular disease: 2023 update. Circulation.

[11] Martínez-González M.A., Gea A., Ruiz-Canela M. (2019). The Mediterranean diet and cardiovascular health: A critical review. Circ. Res..

[12] Micha R., Peñalvo J.L., Cudhea F., Imamura F., Rehm C.D., Mozaffarian D. (2017). Association between dietary factors and mortality from heart disease, stroke, and type 2 diabetes in the United States. JAMA.

[13] Petrelli F., Grappasonni I., Scuri S., Thu N.C.T., Debernardi G., Benni A., Vesprini A., Rocchi R., De Carolis C., Pantanetti P. (2020). Food knowledge of patients at the first access to a Diabetology center. Acta Biomed..

[14] Schulman J.A., Rienzo B.A. (2001). The importance of physicians’ nutrition literacy in the management of diabetes mellitus. Med. Educ. Online.

[15] Dorner B., Friedrich E.K. (2018). Position of the Academy of Nutrition and Dietetics: Individualized nutrition approaches for older adults: Long-term care, post-acute care, and other settings. J. Acad. Nutr. Diet..

[16] von Elm E., Altman D.G., Egger M., Pocock S.J., Gøtzsche P.C., Vandenbroucke J.P. (2007). The Strengthening the Reporting of Observational Studies in Epidemiology (STROBE) statement: Guidelines for reporting observational studies. Lancet.

[17] World Medical Association (2013). World Medical Association Declaration of Helsinki: Ethical principles for medical research involving human subjects. JAMA.

[18] Charan J., Biswas T. (2013). How to calculate sample size for different study designs in medical research?. Indian J. Psychol. Med..

[19] Wang X., Ji X. (2020). Sample size estimation in clinical research: From randomized controlled trials to observational studies. Chest.

[20] IBM Corp. (2015). IBM SPSS Statistics for Windows.

[21] Khera A.V., Emdin C.A., Drake I., Natarajan P., Bick A., Cook N., Chasman D., Baber U., Mehran R., Rader D. (2016). Genetic risk, adherence to a healthy lifestyle, and coronary artery disease. N. Engl. J. Med..

[22] World Health Organization (2022). Noncommunicable Diseases Progress Monitor 2022.

[23] Yang L., Zhang Z., Zhang J., Miao J., Zhang H., Du Y., Ding L. (2026). Stage-specific risk factors of cardiometabolic multimorbidity: A systematic review and meta-analysis from incidence to mortality. J. Clin. Epidemiol..

[24] Trevisan M., Krogh V., Freudenheim J., Blake A., Muti P., Panico S., Farinaro E., Mancini M., Menotti A., Ricci G. (1990). Consumption of olive oil, butter, and vegetable oils and coronary heart disease risk factors. JAMA.

[25] Pimpin L., Wu J.H.Y., Haskelberg H., Del Gobbo L., Mozaffarian D. (2016). Is butter back? A systematic review and meta-analysis of butter consumption and risk of cardiovascular disease, diabetes, and total mortality. PLoS ONE.

[26] Jakobsen M.U., Trolle E., Outzen M., Mejborn H., Grønberg M.G., Lyndgaard C.B., Stockmarr A., Venø S.K., Bysted A. (2021). Intake of dairy products and associations with major atherosclerotic cardiovascular diseases: A systematic review and meta-analysis of cohort studies. Sci. Rep..

[27] Tholstrup T., Raff M., Basu S., Nonboe P., Sejrsen K., Straarup E.M. (2006). Effects of butter high in ruminant trans and monounsaturated fatty acids on lipoproteins, incorporation of fatty acids into lipid classes, plasma C-reactive protein, oxidative stress, hemostasis, and insulin in healthy young men. Am. J. Clin. Nutr..

[28] Vatanparast H., Islam N., Shafiee M., Patil P., Smith A., Whiting S. (2025). Dairy consumption and risk of cardiovascular and bone health outcomes in adults: An umbrella review and updated meta-analyses. Nutrients.

[29] Ruan W., Guo X., Shi S., Li J., Wang T., Liu H., Zhang G., Lin K. (2024). Thyroid function effect on cardiac structure, cardiac function, and disease risk: Evidence of causal associations in European ancestry. Heart Rhythm.

[30] Razvi S., Jabbar A., Pingitore A., Danzi S., Biondi B., Klein I., Peeters R., Zaman A., Iervasi G. (2018). Thyroid hormones and cardiovascular function and diseases. J. Am. Coll. Cardiol..

[31] Critchley J.A., Capewell S. (2003). Mortality risk reduction associated with smoking cessation in patients with coronary heart disease: A systematic review. JAMA.

[32] Tarran R., Barr R.G., Benowitz N.L., Bhatnagar A., Chu H.W., Dalton P., Doerschuk C.M., Drummond M.B., Gold D.R., Goniewicz M.L. (2021). E-Cigarettes and Cardiopulmonary Health. Function.

[33] Shiferaw W.S., Akalu T.Y., Desta M., Kassie A.M., Petrucka P., Aynalem Y.A. (2021). Effect of educational interventions on knowledge of the disease and glycemic control in patients with type 2 diabetes mellitus: A systematic review and meta-analysis of randomized controlled trials. BMJ Open.

[34] Laurenti P., Marziali E., Nachira L., Arcaro P., Villani L., Galasso V., Bruno S., Laurenti P. (2023). A multidisciplinary primary prevention intervention to increase adherence to the Mediterranean diet: A pilot study. BMC Public Health.

[35] Winham D.M. (2009). Culturally tailored foods and CVD prevention. Am. J. Lifestyle Med..

[36] Piwowarczyk E., MacPhee M., Howe J. (2024). Nurses’ role in obesity management in adults in primary healthcare settings worldwide: A scoping review. Healthcare.

