# Supplementary material for: Dietary Habits and Lifestyle Factors Associated with Vascular Diseases: A Case–Control Study
Source: Healthcare (Basel). 2026 Jun 16;14(12):1739. doi: 10.3390/healthcare14121739 (PMC13299332; doi:10.3390/healthcare14121739)
Supplement: Supplementary file 1 [file healthcare-14-01739-s001.zip › healthcare-4310567-supplementary.pdf]

## Supplementary Materials

**Table S1.** STROBE Statement—checklist of items that should be included in reports of case-control studies. *Dietary Habits and Lifestyle Factors Associated with Vascular Diseases: A Case-Control Study.*

| Section / Topic | Item # | STROBE Recommendation | Reported on (Section) |
|---|---|---|---|
| **Title and abstract** | | | |
| **Title and abstract** | **1** | **(a)** Indicate the study's design with a commonly used term in the title or the abstract. | Title; Abstract (Methods) |
| | | **(b)** Provide in the abstract an informative and balanced summary of what was done and what was found. | Abstract |
| **Introduction** | | | |
| **Background / rationale** | **2** | Explain the scientific background and rationale for the investigation being reported. | Sec. 1 (Introduction) |
| **Objectives** | **3** | State specific objectives, including any prespecified hypotheses. | Sec. 1 (final paragraphs) |
| **Methods** | | | |
| **Study design** | **4** | Present key elements of study design early in the paper. | Sec. 2.1 |
| **Setting** | **5** | Describe the setting, locations, and relevant dates, including periods of recruitment, exposure, follow-up, and data collection. | Sec. 2.1; 2.2; 2.4 |
| **Participants** | **6** | **(a)** Give the eligibility criteria, and the sources and methods of case ascertainment and control selection. Give the rationale for the choice of cases and controls. | Sec. 2.2 |
| | | **(b)** For matched studies, give matching criteria and the number of controls per case. | Sec. 2.2 (not applicable – unmatched design) |
| **Variables** | **7** | Clearly define all outcomes, exposures, predictors, potential confounders, and effect modifiers. Give diagnostic criteria, if applicable. | Sec. 2.2; 2.4; 2.5 |
| **Data sources / measurement** | **8** | For each variable of interest, give sources of data and details of methods of assessment (measurement). Describe comparability of assessment methods if there is more than one group. | Sec. 2.4 |
| **Bias** | **9** | Describe any efforts to address potential sources of bias. | Sec. 2.5; Sec. 4 (illness effect / protopathic bias) |
| **Study size** | **10** | Explain how the study size was arrived at. | Sec. 2.3 |
| **Quantitative variables** | **11** | Explain how quantitative variables were handled in the analyses. If applicable, describe which groupings were chosen and why. | Sec. 2.5 |
| **Statistical methods** | **12** | **(a)** Describe all statistical methods, including those used to control for confounding. | Sec. 2.5 |
| | | **(b)** Describe any methods used to examine subgroups and interactions. | Sec. 2.5 (not applicable) |
| | | **(c)** Explain how missing data were addressed. | Sec. 2.5 (listwise deletion) |

| Section / Topic | Item # | STROBE Recommendation | Reported on (Section) |
|---|---|---|---|
| | | **(d)** If applicable, explain how matching of cases and controls was addressed. | Sec. 2.5 (not applicable – unmatched) |
| | | **(e)** Describe any sensitivity analyses. | Sec. 2.5 (collinearity / VIF; model fit) |
| **Results** | | | |
| **Participants** | **13** | **(a)** Report numbers of individuals at each stage of study—e.g. numbers potentially eligible, examined for eligibility, confirmed eligible, included in the study, completing follow-up, and analysed. | Sec. 3.1; Table 1 |
| | | **(b)** Give reasons for non-participation at each stage. | Sec. 2.2 (exclusion criteria) |
| | | **(c)** Consider use of a flow diagram. | Not applicable |
| **Descriptive data** | **14** | **(a)** Give characteristics of study participants (e.g. demographic, clinical, social) and information on exposures and potential confounders. | Sec. 3.1–3.5; Tables 1–5 |
| | | **(b)** Indicate number of participants with missing data for each variable of interest. | Tables 1–4 (denominators in parentheses) |
| **Outcome data** | **15** | Report numbers in each exposure category, or summary measures of exposure. | Sec. 3.3–3.4; Tables 3–4 |
| **Main results** | **16** | **(a)** Give unadjusted estimates and, if applicable, confounder-adjusted estimates and their precision (e.g. 95% confidence interval). Make clear which confounders were adjusted for and why they were included. | Sec. 3.6; Table 6 |
| | | **(b)** Report category boundaries when continuous variables were categorized. | Sec. 2.5; Table 6 (ferritin per 10 ng/mL; age per year) |
| | | **(c)** If relevant, consider translating estimates of relative risk into absolute risk for a meaningful time period. | Not applicable |
| **Other analyses** | **17** | Report other analyses done—e.g. analyses of subgroups and interactions, and sensitivity analyses. | Sec. 3.6 (Hosmer–Lemeshow; Nagelkerke $R^2$) |
| **Discussion** | | | |
| **Key results** | **18** | Summarise key results with reference to study objectives. | Sec. 4 (Discussion, opening) |
| **Limitations** | **19** | Discuss limitations of the study, taking into account sources of potential bias or imprecision. Discuss both direction and magnitude of any potential bias. | Sec. 4 (illness effect, reverse causality, non-standardized sampling) |
| **Interpretation** | **20** | Give a cautious overall interpretation of results considering objectives, limitations, multiplicity of analyses, results from similar studies, and other relevant evidence. | Sec. 4 |
| **Generalisability** | **21** | Discuss the generalisability (external validity) of the study results. | Sec. 4 (Central Anatolian context) |

| Section / Topic | Item # | STROBE Recommendation | Reported on (Section) |
|---|---|---|---|
| **Other information** | | | |
| **Funding** | **22** | Give the source of funding and the role of the funders for the present study and, if applicable, for the original study on which the present article is based. | Funding statement (end of article) |

**Note:** An Explanation and Elaboration article discusses each checklist item and gives methodological background and published examples of transparent reporting. The STROBE checklist is best used in conjunction with this article (freely available on the Web sites of PLoS Medicine at http://www.plosmedicine.org/, Annals of Internal Medicine at http://www.annals.org/, and Epidemiology at http://www.epidem.com/). Information on the STROBE Initiative is available at http://www.strobe-statement.org.

*Section numbers refer to the manuscript headings; exact page and line numbers should be inserted according to the final journal layout prior to submission.*

**Table S2.** Content and structure of the study questionnaire.

STUDY INSTRUMENT · SURVEY QUESTIONNAIRE

# Content and structure of the study questionnaire

*Comparison of nutrition and lifestyle in vascular disease: the Yozgat example — a self-administered questionnaire covering sociodemographic, clinical, behavioural, familial and dietary domains.*

## 1 Sociodemographic & occupational profile

- Age
  *years*
- Sex
  *female / male*
- Weight; Height
  *kg / cm*
- Occupation
  *civil servant · worker · self-employed · homemaker · unemployed*
- Workplace field of activity
  *open response (e.g., chemical, health, agriculture)*
- Workplace hazard class
  *low · hazardous · very hazardous · unknown*
- Job / task performed
  *open response (e.g., welder, painter, clerk)*
- Years at current workplace
  *0–2 · 2–5 · 5–10 · >10 years*
- Monthly income
  *4 bands (≤2000 → ≥10000 TL)*
- Education level
  *8 levels (illiterate → postgraduate)*
- Place of residence
  *rural / urban*
- Marital status
  *married · single · divorced · widowed*

## 2 Personal medical history

- Chronic disease present
  *yes / no*
- Type of chronic disease
  *hypertension · diabetes · coronary heart disease · stroke · renal · asthma/dyspnoea · hyperlipidaemia · psychiatric · other*
- Surgical history
  *none · cardiac · vascular · renal · abdominal · pulmonary · other*
- Regular medication use
  *open response*
- Frequency of mental fatigue
  *always · weekly · monthly · rarely · never*

## 3 Lifestyle behaviours

- Smoking status
  *never · former · current (cigarettes/day)*
- Alcohol use
  *no / yes (days/month)*
- Regular physical exercise
  *daily · 3–4×/wk · 1×/wk · none*

## 4 Family history (1st–2nd-degree relatives)

- Family chronic disease present
  *yes / no*
- Type of family disease
  *hypertension · diabetes · coronary heart disease · stroke · renal · asthma/dyspnoea · hyperlipidaemia · psychiatric · other*

## 5 Dietary habits & nutrition knowledge

- Food supplement use
  *yes / no*
- Red meat
  *2–3×/wk · 1–2×/wk · 1–2×/mo · rarely · never*
- Fish & seafood
  *2–3×/wk · 1–2×/wk · 1–2×/mo · rarely · never*
- Fruit & vegetables
  *2–3×/wk · 1–2×/wk · 1–2×/mo · rarely · never*
- White meat (poultry)
  *2–3×/wk · 1–2×/wk · 1–2×/mo · rarely · never*
- Milk & dairy products
  *2–3×/wk · 1–2×/wk · 1–2×/mo · rarely · never*
- Eggs
  *2–3×/wk · 1–2×/wk · 1–2×/mo · rarely · never*
- Legumes
  *2–3×/wk · 1–2×/wk · 1–2×/mo · rarely · never*
- Fast food
  *2–3×/wk · 1–2×/wk · 1–2×/mo · rarely · never*
- Junk food (chocolate, chips...)
  *2–3×/wk · 1–2×/wk · 1–2×/mo · rarely · never*
- Canned products
  *2–3×/wk · 1–2×/wk · 1–2×/mo · rarely · never*
- Mineral water consumption
  *6-level scale (daily 1–3 btl → never)*
- Daily sugar use
  *none · low · normal · high*
- Daily salt use
  *saltless · low · normal · heavily salted*
- Cooking fat type (multiple)
  *animal fat · margarine · butter · sunflower · corn · olive oil*
- Eating speed
  *slow · normal · fast · very fast*
- Food-additive label checking
  *never · sometimes · always*
- Monthly per-capita food spending
  *open response*
- Awareness: mineral water raises BP
  *yes / no*
- Self-rated healthy-eating habit
  *scored 1–10*

Food-frequency items (red meat, fish, fruit & vegetables, poultry, dairy, eggs, legumes, fast food, junk food, canned products) used a five-point ordinal scale: 2–3×/week · 1–2×/week · 1–2×/month · rarely · never. Italic text indicates response options; "open response" denotes free-text fields. Original instrument administered in Turkish; items translated for presentation.
